# Mycobacterium avium Complex Lung Disease Complicated With Antiglomerular Basement Membrane Disease: A Case Report

**DOI:** 10.7759/cureus.32192

**Published:** 2022-12-05

**Authors:** Shoma Hirota, Kosaku Komiya, Yukiko Takeno, Kotaro Miyazaki, Yuichi Tokunaga, Kazufumi Hiramatsu, Jun-ichi Kadota

**Affiliations:** 1 Respiratory Medicine and Infectious Diseases, Oita University Faculty of Medicine, Yufu, JPN; 2 Respiratory Medicine, Shinbeppu Hospital, Beppu, JPN

**Keywords:** clarithromycin, corticosteroid, anti-gbm antibody, anti-gbm disease, mycobacterium avium complex

## Abstract

While both *Mycobacterium avium *complex (MAC) lung diseases and antiglomerular basement membrane (anti-GBM) antibody disease may cause hemoptysis, no case presenting hemoptysis having both diseases has been reported. A woman in her 80s was admitted due to hemoptysis with acute respiratory failure. MAC was isolated from her sputum, and a positive report for anti-GBM antibody was confirmed in screening for hematuria. This patient has been successfully treated with systemic corticosteroid therapy followed by combination chemotherapy against MAC. Although anti-GBM disease is a rare condition, screening might be recommended in case of uncontrollable hemoptysis as MAC lung disease with hematuria.

## Introduction

Hemoptysis is caused by various pulmonary diseases, including infections, autoimmune diseases, and malignancy [1]. *Mycobacterium avium* complex (MAC) is a major pathogen that induces chronic infectious disease with hemoptysis, and its morbidity and mortality are increasing worldwide [2]. Antiglomerular basement membrane (anti-GBM) disease is a rare autoimmune disease characterized by severe alveolar hemorrhage and glomerulonephritis [3]. Hemoptysis is developed in 37% of MAC lung diseases and 28% of anti-GBM diseases [4,5], whereas the incidence of MAC lung disease and anti-GBM disease are estimated to be 15/100,000 and 0.5/1,000,000, respectively. Consequently, complications attributed to both diseases are rare. In this manuscript, we report a case presenting hemoptysis in MAC lung disease complicated with anti-GBM antibody disease and discuss possible interactions between both diseases to develop an effective treatment strategy.

## Case presentation

A woman in her 80s visited our hospital due to hemoptysis persisting since the day before. She coughed out approximately 30 mL of blood and then required oxygen inhalation. Although she had a history of hemoptysis two years before admission, no causative mechanism was detected. The physical examination revealed a body temperature of 37.5°C, a percutaneous oxygen saturation (SpO2) of 88% without supplemental oxygenation, blood pressure of 161/73 mmHg, and a heart rate of 88 beats/min. The laboratory blood tests showed a white blood cell count of 5,600/μL, hemoglobin level of 10.0 g/dL, C-reactive protein level of 0.45 mg/dL, slightly elevated blood urea nitrogen level of 25.3 mg/dL, and serum creatinine level of 0.64 mg/dL. The biomarkers of the coagulation system including D-dimer remained within normal range. The urinary test showed microscopic hematuria but not proteinuria or pyuria. Urine culture was negative.

**Figure 1:**
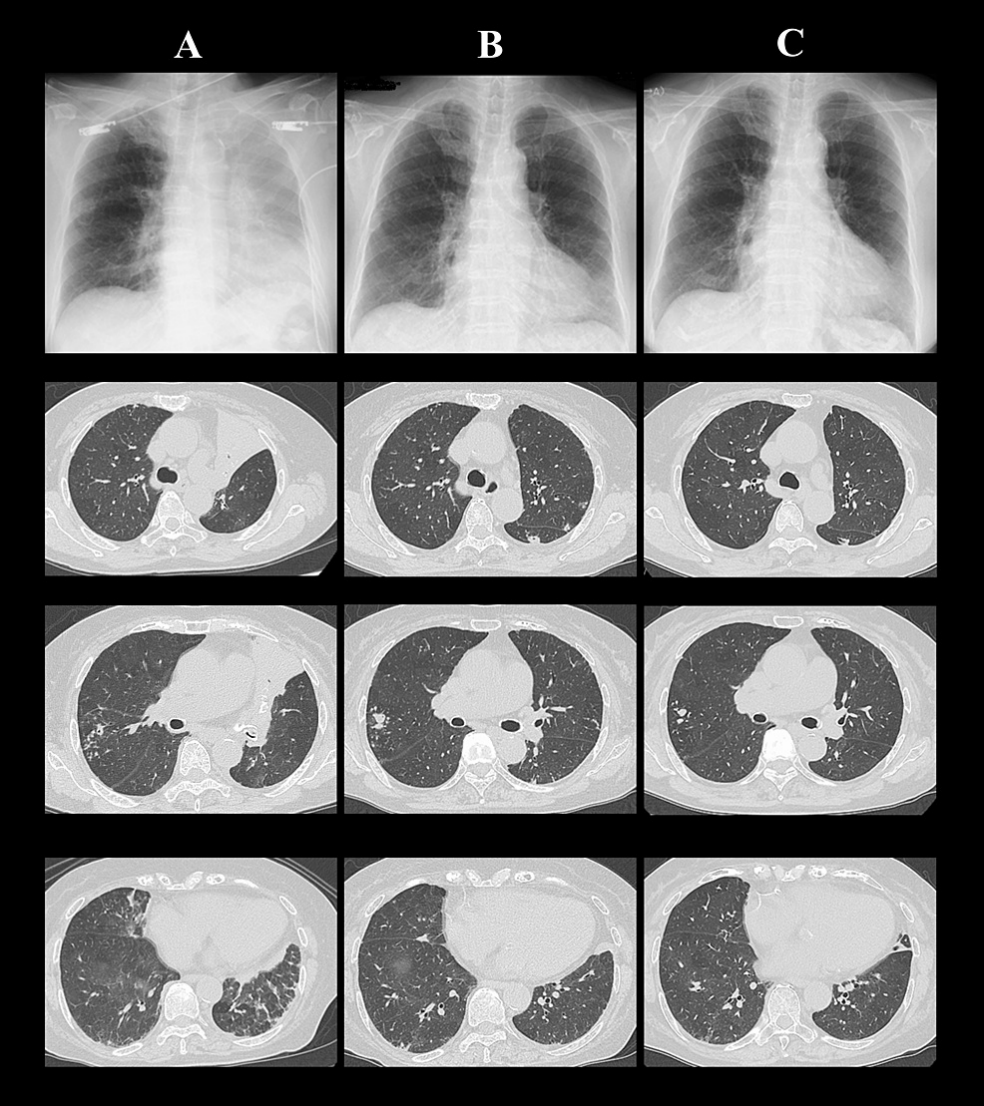
Chest X-ray and computed tomography at the upper lobes, middle lobes, and lower lobes (A) upon admission, (B) after 1.5 months from admission, and (C) after six months from admission

Chest X-rays and CT were obtained for the upper, middle, and lower lobes at the time of admission and during the course of treatment (Figures 1A-1C). A chest CT revealed atelectasis in the left upper lobe, multifocal glass opacities, and centrilobular nodules with bronchiectasis in the right middle lobe (Figure 1A). We suspected alveolar hemorrhage and administered tranexamic acid (1000 mg/day), carbazochrome sodium sulfonate (50 mg/day), and nicardipine hydrochloride (48 mg/day) to the patient intravenously. The bronchoscopic examination was not conducted at that time. However, after three hours, her respiratory status worsened, which required 7 L/min of oxygen to maintain a SpO2 above 90%. A sputum culture obtained upon admission revealed *M. avium* but not *M. tuberculosis* isolation. Since microscopic hematuria was observed, we suspected nephritis complicated with MAC lung disease considering the refractory respiratory failure. While the cytoplasmic and perinuclear antineutrophil antibodies were both negative, the anti-GBM antibody measured by chemiluminescent enzyme immunoassay (Life Sciences Company, Nagano, Japan) was elevated (4.5 U/ml > 3.0 U/ml). We considered renal biopsy but it was suspended because of normal renal function. No elevation of rheumatoid factor, anti-cyclic citrullinated peptide antibody, and thyroid hormone were confirmed. While the anti-MAC antibody was positive (>10.0 U/mL), the Interferon-γ release assay (T-SPOT.TB test, Oxford Immunotec, Oxford, UK) was found to be negative.

On day five of admission, we treated her with high-dose methylprednisolone (1,000 mg/day) for three days against refractory respiratory failure considering her anti-GBM disease. As renal function was not deteriorating, we did not add immunosuppressants such as cyclophosphamide. On day 23, her respiratory status gradually improved, and she no longer required supplemental oxygenation. The microscopic hematuria also disappeared on day eight. Since atelectasis in the left upper lobe remained not to be released, we performed a bronchoscopy examination on day 27. The left upper segmental bronchus was found to be obstructed with blood clots. After removing the endobronchial blood clots, the atelectasis was confirmed to be improved. The dose of corticosteroids had been reduced to 15 mg of prednisolone, and the patient was discharged on day 42 after confirming the decreased level of anti-GBM antibodies to 3.3 U/ml.

On day 16, *M. avium* but not *M. tuberculosis* was continuously isolated from her sputum, which was obtained on different days, following the diagnostic criteria of the American Thoracic Society/the Infectious Diseases Society of America (ATS/IDSA) for the treatment of nontuberculous mycobacterial pulmonary disease. On day 84, centrilobular nodules expanded and a cavitary lesion was observed on chest CT when reducing the dose of corticosteroids (Figure 1B). Since the quantity of *M*. *avium* in the sputum was found to increase from 1+ to 3+, we initiated the treatment with the combination of 450 mg/day of rifampicin, 500 mg/day of ethambutol, and 800 mg/day of clarithromycin for MAC lung disease. Considering the drug interactions between prednisolone and rifampicin, we increased the dose of prednisolone from 5 mg to 10 mg and continue the same dose to date.

After six months from admission, the patient remains stable without recurrence of hemoptysis and microscopic hematuria, and lung involvement has improved (Figure 1C). Upon further genetic examination, we tested her human leukocyte antigen (HLA) class, indicating that she had serotype HLA DR4, which is associated with anti-GBM antibody disease. However, whether disease prognosis varies by HLA type remains uncertain.

## Discussion

Herein, we report a case of MAC lung disease complicated with anti-GBM disease, presenting hemoptysis with refractory respiratory failure. The patient was treated with steroidal therapy, following multidrug chemotherapy for MAC lung disease, because its exacerbation was suspected.

No case of coexisting MAC lung disease and anti-GBM disease has been reported yet, probably because anti-GBM disease is quite rare and anti-GBM antibody is less commonly tested. First, the pathological interactions between both diseases need to be discussed. Apart from the incidental occurrence of these diseases, MAC infection may have induced anti-GBM disease or vice versa. As a hypothesis of anti-GBM disease development, proceeding damages may lead to the exposure of the antigen epitope of anti-GBM antibody on the glomerular or alveolar basement membranes [6]. MAC infection causes chronic inflammation in small airways and alveoli, which is observed to have a tree-in-bud appearance with bronchiectasis on chest CT. In this regard, MAC infection possibly leads to the exposure of alveolar basement membranes, which induces anti-GBM antibody production. On the contrary, anti-GBM disease may distort lung structure through alveolar basement membrane damage. Patients with lung tissue destruction, including chronic obstructive pulmonary disease and bronchiectasis, are at a high risk of MAC lung disease. When it is hypothesized that anti-GBM disease proceeds before MAC lung disease, the impairment of the alveolar basement membrane that resulted from anti-GBM disease would contribute to MAC infection [7].

HLA DR15 and DR4 are reported to be associated with the development of anti-GBM antibody disease [8]. The patient was found to have serotype HLA DR4, which would have affected the progression of her disease. Considering the chest CT findings showing bronchiectasis upon her initial visit, which denotes persistent airway inflammation, MAC infection may antecede before the development of anti-GBM disease [9]. Furthermore, microscopic hematuria was first detected upon admission even though she has taken an annual health check-up. This might refer to the recent development of anti-GBM antibody diseases along with the background of serotype HLA DR4 and MAC infection.

## Conclusions

We report a case of MAC lung disease complicated with the anti-GBM disease. The percentage of positive results for anti-GBM antibodies among patients with MAC lung diseases has not yet been determined. If the positivity among MAC lung diseases is higher than that in the general population, MAC infection would interact with the anti-GBM disease. Although anti-GBM disease is a rare condition, in the case of uncontrollable hemoptysis such as MAC lung disease, a urinary test might be recommended to screen for the complications of nephritis.
